# Comparative effects of single-task and dual-task acute moderate-intensity exercise on cortisol and cognitive performance

**DOI:** 10.3389/fpsyg.2025.1700931

**Published:** 2025-12-18

**Authors:** Zhongshu Shao, Jiazheng Peng, Minggang Luo, Talifu Zikereya, Jinliang Chen, Kaixuan Shi

**Affiliations:** 1 Department of Physical Education, China University of Geosciences, Beijing, China; 2 School of Physical Education, University of Science and Technology Beijing, Beijing, China

**Keywords:** acute exercise, cortisol, cognitive functions, orienteering, dual-task, HPA axis, memory, executive function

## Abstract

**Background:**

Acute physical exercise elicits neuroendocrine responses that may transiently modulate cognitive performance. While moderate-intensity single-task exercise (e.g., running) has been shown to improve cognition, the acute cognitive and hormonal effects of dual-task exercise, which involves cognitive load, remain underexplored.

**Objectives:**

This study aims to compare the effects of moderate-intensity single-task (5-km run) and dual-task (orienteering) exercise on salivary cortisol levels and cognitive performance in healthy young adults.

**Methods:**

Twenty-one male college students (age: 20.57 ± 2.57 years) participated in a randomized crossover design. Each participant completed both exercise conditions. Salivary cortisol and cognitive performance (assessed via 2-Back, Mental Rotation, and Bells visual search tests) were measured before and after exercise. Exercise intensity was standardized using VO₂max, and heart rate was continuously monitored.

**Results:**

Orienteering elicited a significant increase in salivary cortisol (*p <* 0.01), whereas 5-km run did not. Both exercises significantly improved reaction times (RTs) on the 2-Back and Mental Rotation tasks (*p <* 0.01), though no significant improvements in task accuracy were observed (*p* > 0.05). Performance on the Bells task improved post-exercise but showed no correlation with cortisol changes. Increases in cortisol were positively associated with RTs and accuracy improvements on working memory and spatial tasks, but not with visual search.

**Conclusion:**

Dual-task exercise induced stronger neuroendocrine responses and broader cognitive benefits compared to single-task running. These findings suggest that integrating cognitive demands into exercise routines may enhance acute cortisol-driven facilitation of executive functions, highlighting the importance of such exercises in programs targeting cognitive and brain health.

## 1 Introduction

Acute exercise is a potent physiological stressor that transiently activates the sympathetic–adreno-medullary system and the hypothalamic–pituitary–adrenal (HPA) axis, leading to transient increases catecholamines and cortisol secretion (Wunsch et al., 2019; Russell and Lightman, 2019). These hormonal changes facilitate rapid alterations in brain function, as circulating cortisol rapidly crosses the blood–brain barrier and binds densely to receptors in the prefrontal cortex and hippocampus, which are involved in executive functioning, working memory, and visuospatial processing (Caplin et al., 2021; Lupien et al., 2009). These cognitive domains are crucial for both athletic performance and daily tasks, including decision-making and spatial orientation (Wilke et al., 2020).

While chronic exposure to elevated stress hormones is known to impair cognition, acute increases in cortisol resulting from moderate-intensity exercise may enhance cognitive performance by boosting arousal and attentional resources (Basso and Suzuki, 2017; Shields et al., 2019). Moderate-intensity activities like submaximal running have been shown to improve processing speed and reaction times (RTs) (Suwabe et al., 2018; Gronwald et al., 2018). Conversely, high-intensity or prolonged exercise has been associated with excessive cortisol release, which may impair memory and learning performance (Bermejo et al., 2022).

Beyond exercise intensity, the cognitive demands embedded within the exercise itself also influence neuroendocrine and cognitive outcomes (Kraemer and Kraemer, 2023). Dual-task activities, which involve simultaneous physical exertion and cognitive engagement, have produced mixed results in terms of cognitive performance (Gallou-Guyot et al., 2020; Budde et al., 2008). Some studies report cognitive interference and slower RTs, possibly due to resource competition (Davis et al., 2022), while others find that dual-task exercise facilitates cognitive performance, despite elevated cortisol levels (Pellegrini-Laplagne et al., 2022). Although chronic dual-task training has been linked to improvements in visuospatial accuracy (ACC) and working memory in youth (Koutsandréou et al., 2016), the acute effects of such exercise on cortisol and cognition in adults remain underexplored (Becker et al., 2023).

Discrepancies in previous studies arise from varying definitions of exercise intensity, modality, and cognitive assessments. Objective measures such as percentage of maximal oxygen uptake (%VO₂max) or lactate thresholds are preferable to subjective descriptors (Budde et al., 2015; Gronwald et al., 2019). Additionally, few studies have examined the combined effects of dual-task exercise and cortisol on immediate cognitive performance using ecologically valid paradigms.

To address these gaps, we conducted a randomized, crossover study comparing two moderate-intensity exercise modes commonly used in university sport settings: (1) a steady-state 5-km run (single-task), and a short-distance orienteering course requiring simultaneous navigation (dual-task). Salivary cortisol and RTs were measured before and after exercise.

The study addressed three key questions: (1) Does dual-task exercise elicit a greater acute cortisol response than single-task running? (2) Do both exercise modes improve post-exercise RTs relative to baseline, and is the greater improvement associated with the larger cortisol increases? (3) Does the magnitude of cortisol change predict improvements in RTs, consistent with a stress-related cognitive facilitation effect?

By answering these questions, this study aims to inform future randomized trials and provide practical insights for integrating physical and cognitive training in athletic and educational settings.

## 2 Methods

### 2.1 Participants

Twenty-four male college students were recruited from a university sports club located in Beijing. Inclusion criteria were: (1) right-handedness, (2) at least 1 year of orienteering experience, (3) no history of neurological, cardiovascular, or metabolic diseases, (4) non-smoking status, and (5) no current use of medication. *A priori* power analysis (*α* = 0.05, power = 0.80) was performed using PASS 2021, indicating that a sample size of 19 would be sufficient to detect a medium effect size (Cohen’s d = 0.65) for the primary outcome. To accommodate potential dropouts, 24 participants were enrolled. All participants provided written informed consent. The protocol was approved by the Ethics Committee of China University of Geosciences (CUGB-EC-2023002; approved on 18 March 2023).

### 2.2 Experimental design

A randomized, within-subject crossover design was used in this study. Participants first completed baseline assessments and were then randomly assigned to two groups (A and B). In the first week, participants completed either an orienteering task or a 5-km run, and task assignments were reversed in the second week. All sessions were scheduled between 09:00 and 11:00 to control for circadian fluctuations in cortisol levels. Before each session, participants completed a standardized warm-up consisting of 5 min of light jogging and dynamic mobility exercises. Following exercise, participants rested in a seated position for 2 min before undergoing post-exercise assessments. There was no separate resting control day; baseline (PRE) measures were collected immediately before each exercise session. In the single-task condition, participants ran 12.5 laps (5-km run) on a 400-m outdoor track at 60–70% of their maximal oxygen uptake (VO₂max), which corresponded to 64–76% of their individually measured maximal heart rate (HRmax) (Garber et al., 2011; Wohlwend et al., 2017). In the dual-task condition, participants completed a 5-km orienteering course with 27 checkpoints distributed across mixed terrain in a public park while maintaining the same target heart rate (HR) zone as in the single-task condition. Heart rate (HR) was continuously monitored using a Polar H10 chest strap (Polar Electro Oy, Kempele, Finland). For orienteering, GPS data were recorded using a wrist device synchronized with the HR monitor. Time spent within the target HR zone (64–76% HRmax) was calculated for each condition, and participants were required to maintain at least 80% of total exercise time within the target zone (reported in Table 1).

**Table 1:** Basic characteristics of the participants (Mean *=* SD; *n =* 21).

Variables		Mean (SD)
Age (year)		20.57 ± 2.57
Weight (kg)		64.00 ± 7.68
Height (m)		174.14 ± 3.85
Body fat (%)		13.05 ± 4.21
BMI (kg/m^2^)		21.08 ± 2.39
VO_2max_ (mL/kg/min)		56.04 ± 8.46
HR (bpm)	Dual-task	157.88 ± 19.11
	Single-task	158.00 ± 14.47
RPE	Dual-task	14.67 ± 0.83
	Single-task	13.88 ± 0.76
Time in target HR zone (%)	RUN ORI	88.7 ± 6.9 85.2 ± 8.3

Summary of participant characteristics, including age, weight, height, body fat percentage, BMI, VO_2max_, HR during orienteering and 5-km run, and RPE for both activities. BMI, Body Mass Index; HR, Heart Rate; RPE, Borg Rating of Perceived Exertion.

### 2.3 Maximal oxygen uptake test

Maximal oxygen uptake (VO₂max) was assessed on a motorized treadmill (Cosmed, Rome, Italy) using a standardized incremental protocol, with calibration performed before each session. Participants wore a breath-by-breath gas analyzer (Cosmed K5, Rome, Italy) and a chest-strap heart-rate monitor (Polar H10, Polar Electro Oy, Kempele, Finland). Speed started at 8.0 km·h^−1^ (0% incline) and increased by 1 km·h^−1^ every 3 min until volitional exhaustion; HR and oxygen uptake were continuously recorded (Hawley and Noakes, 1992). According to the guidelines of the American College of Sports Medicine (ACSM), the criterion for exhaustion was set as: VO₂ plateau (< 2.1 mL·kg^−1^·min^−1^ despite increasing workload), respiratory exchange ratio ≥ 1.10, peak HR ≥ 95% age-predicted maximum (220 − age) or ≥ 180 bpm, rating of perceived exertion (Borg 6–20) ≥ 19, and voluntary termination despite verbal encouragement. VO₂max was the highest 20-s rolling mean of oxygen uptake; HRmax was the peak HR during the test.

### 2.4 Saliva cortisol collection

Salivary cortisol was sampled at PRE and POST between 09:00 and 11:00 to minimize circadian variation. Participants recorded wake time, and the wake-to-sampling interval was calculated (mean ± SD: 2.5 ± 0.8 h). Before sampling, participants refrained from eating, drinking (except water), smoking, and brushing teeth for ≥ 30 min. Unstimulated saliva was collected by passive drool into sterile polypropylene tubes kept on ice, centrifuged at 3,000 × g for 10 min at 4 °C, aliquoted into 0.5-mL cryovials and stored at −80 °C (single thaw before assay). Cortisol was measured in duplicate using ELISA (Xinyu Biotech, XY-9H0641, Beijing, China) with high/low QC per run; sensitivity 0.1 ng·mL^−1^, dynamic range 0.1–50 ng·mL^−1^; intra−/inter-assay CVs 4.8%/6.1%.

### 2.5 Cognitive test data collection

Immediately after saliva collection, participants completed three cognitive tasks in randomized order using parallel versions, with five practice trials per task before the first assessment. Reaction time (RT) and accuracy (ACC) were recorded for computerized tasks; RT outliers ± 3 SD from each participant’s mean were excluded. The 2-Back task comprised 120 trials (target:non-target = 30%:70%; 36/84) with variable ISI of 1,000–2,000 ms; responses via designated keys; outcomes: mean RT (ms), accuracy (% correct) (Lunardini et al., 2020). The Mental Rotation task included 48 pairs of 3D Figures (24 same, 24 different) at 0°, 50°, 100°, 150° (12 pairs/angle), displayed until response or 7,500 ms; outcomes: mean RT and accuracy. The Bells Visual Search test required identifying/circling as many target bells as possible within 30 s on a standardized paper grid (35 targets; max score = 35); distributions were summarized descriptively (mean ± SD, range, median).

### 2.6 Statistical analysis

**Figure 1:**
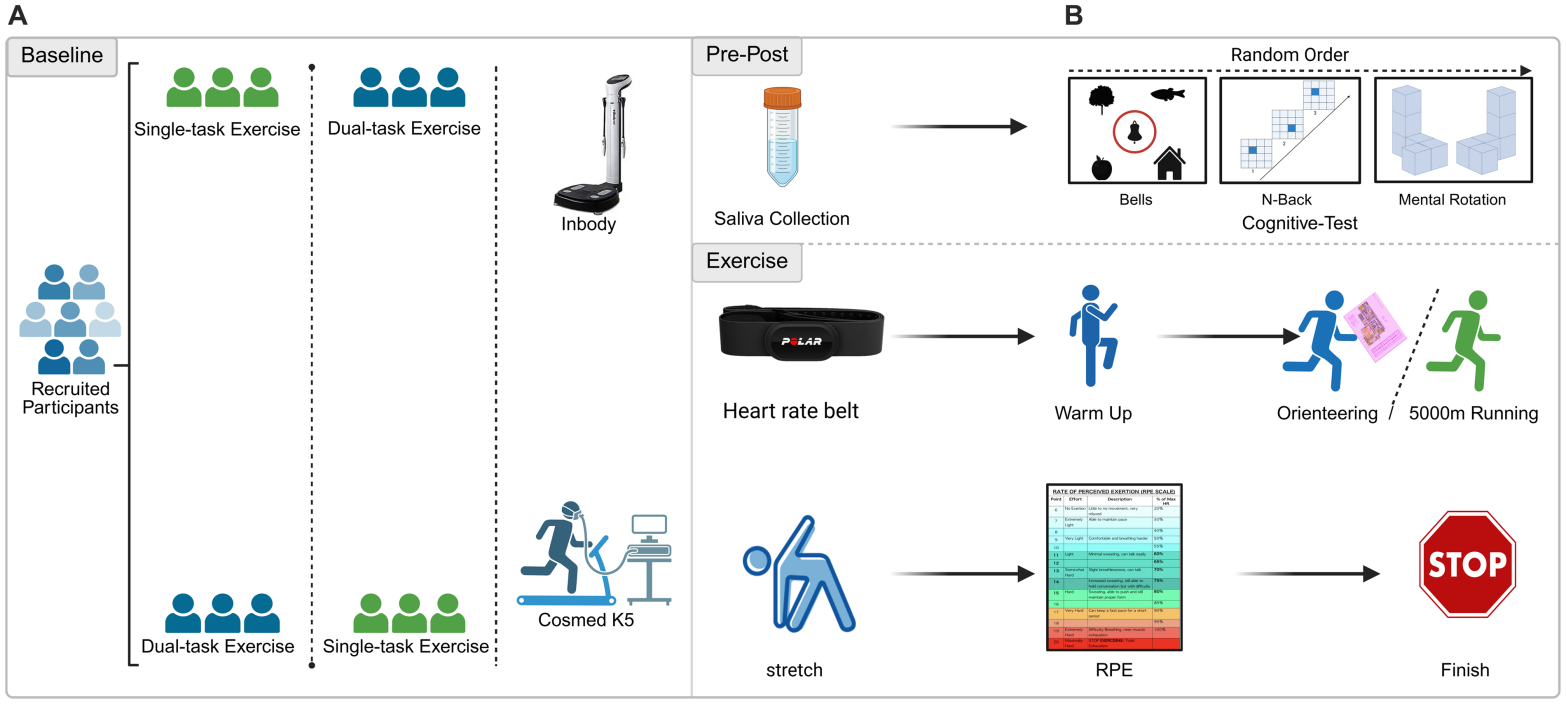
Experimental design. **(A)** Twenty-one participants completed both single-task (5-km run, RUN) and dual-task (orienteering, ORI) conditions in a randomized crossover order. Baseline assessments included body composition (InBody) and VO₂max (Cosmed K5). **(B)** In each session, saliva and cognitive tests (Bells, 2-Back, Mental Rotation) were collected at baseline/pre-exercise (RUN-PRE/ORI-PRE) and immediately post-exercise (RUN-POST/ORI-POST). Participants performed a standardized warm-up, followed by either exercise mode. HR was monitored (Polar H10), and RPE was recorded post-exercise.

The primary statistical model was a 2 × 2 repeated-measures ANOVA (RM-ANOVA) with Condition (RUN vs. ORI) and Time (PRE vs. POST) as within-subjects factors. For cortisol, we first tested the Condition × Time interaction, followed by Bonferroni-corrected paired comparisons. For cognitive outcomes (baseline, RUN-POST, ORI-POST), one-way repeated-measures ANOVA with Bonferroni-corrected *post-hoc* tests was used. For non-normal data, Friedman tests and Wilcoxon signed-rank tests with Bonferroni correction were applied. Effect sizes were reported as partial eta-squared (*η_p_*^2^) for ANOVA and Cohen’s d for pairwise comparisons. Two-tailed *α* = 0.05 (Figure 1).

## 3 Results

### 3.1 Participants

Of the 24 individuals initially recruited, 21 completed all three visits and were included in the final analysis (Table 1). The final sample had a mean age of 20.57 ± 2.57 years, mean weight of 64.00 ± 7.68 kg, height of 174.14 ± 3.85 cm, body mass index (BMI) of 21.08 ± 2.39 kg·m^−2^, and maximal oxygen uptake (VO₂max) of 56.04 ± 8.46 mL·kg^−1^·min^−1^. No adverse events were observed or reported during either of the exercise modalities.

### 3.2 Cortisol

**Figure 2:**
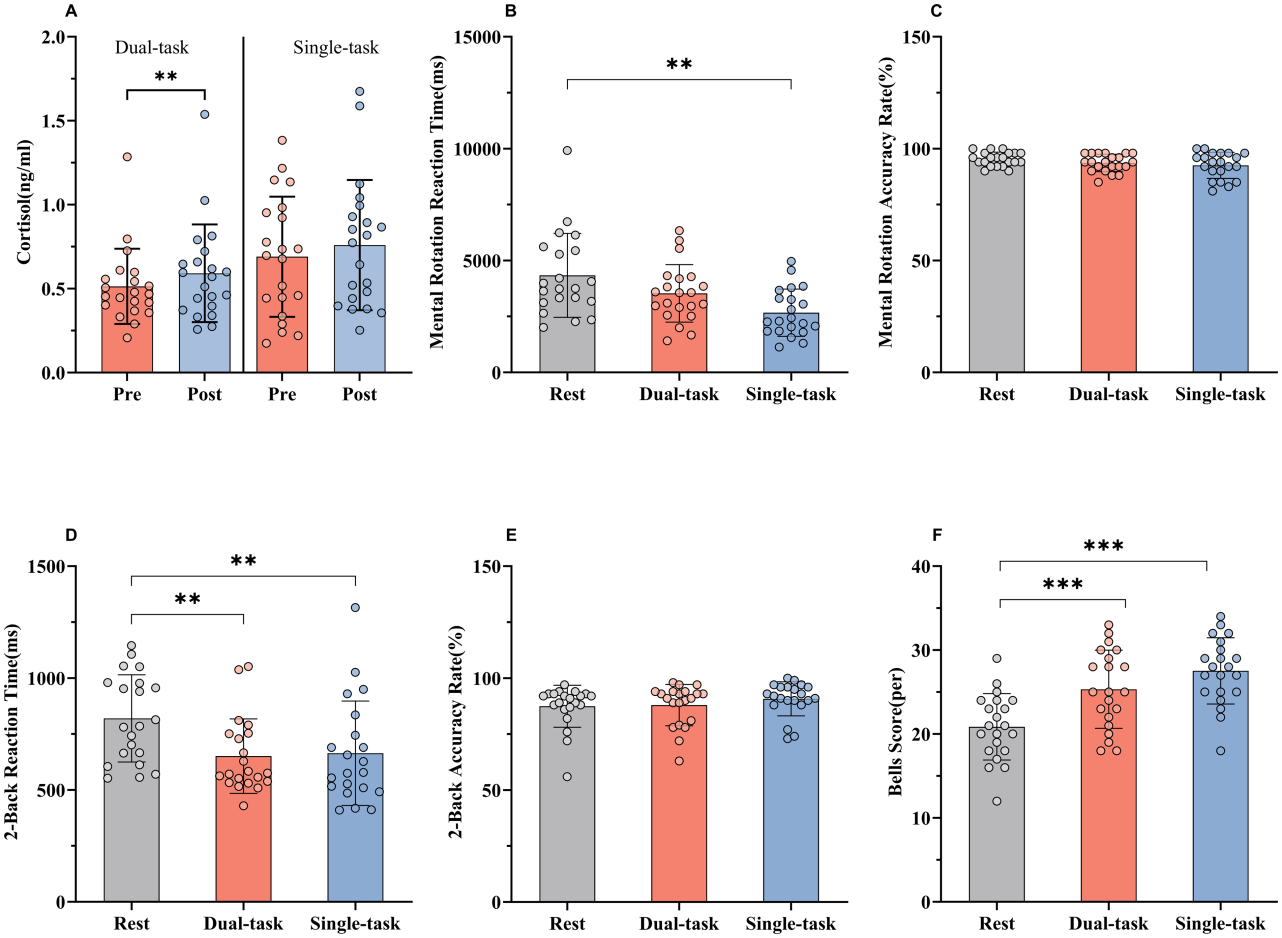
Cortisol levels and cognitive function test results. **(A)** Changes in salivary cortisol from baseline/pre-exercise (RUN-PRE/ORI-PRE) to post-exercise (RUN-POST/ORI-POST). **(B,C)** Mental rotation RTs and ACC across baseline, RUN-POST, and ORI-POST. **(D,E)** 2-Back RTs and ACC across baseline, RUN-POST, and ORI-POST. **(F)** Bells score across baseline, RUN-POST, and ORI-POST. *p < 0.05; **p < 0.01; ***p < 0.001.

As shown in Figure 2A, orienteering (dual-task) elicited a significant increase in salivary cortisol levels from pre- to post-exercise (Pre: 0.513 ± 0.224 ng·mL^−1^; Post: 0.592 ± 0.291 ng·mL^−1^; t = 3.814, *p* = 0.001), indicating a robust endocrine response.

In contrast, 5-km run (single-task) did not result in a significant change in cortisol concentrations between pre- and post-exercise (*p* > 0.05), suggesting a more stable endocrine response under this condition.

### 3.3 Cognitive function

#### 3.3.1 Mental rotation

A repeated-measures ANOVA was performed to assess mental rotation performance across conditions (Figures 2B,C). A significant main effect of condition was observed for RTs (*F* = 7.986, *p* = 0.001, R^2^ = 0.285). Post-hoc multiple comparisons revealed a significant reduction in RT following the 5-km run compared to baseline (p = 0.001, t = 3.996). Although RT also decreased after the orienteering relative to baseline, this difference did not reach statistical significance (*p* = 0.117, t = 1.932).

ACC was evaluated using a one-way ANOVA, which showed no significant differences between conditions (*F* = 2.916, *p* = 0.117, R^2^ = 0.11). Pairwise comparisons confirmed that neither orienteering (*p* = 0.264, t = 1.498) nor the 5-km run (*p* = 0.042, t = 2.389) significantly affected ACC compared to baseline.

#### 3.3.2 2-Back test

The RT and ACC in the 2-Back task were analyzed across three conditions - baseline, orienteering, and 5-km run using one-way ANOVA (Figures 2D,E). A significant effect of condition was found for RT (*F* = 10.73, *p <* 0.001, R^2^ = 0.349). Post-hoc comparisons revealed significantly faster RTs following both the orienteering (*p <* 0.001, t = 4.160) and the 5-km run (*p <* 0.001, t = 3.846) compared to baseline.

For accuracy rate, no significant differences were observed among conditions (*F* = 2.128, *p* = 0.132, R^2^ = 0.096). Pairwise comparisons confirmed that ACC did not significantly differ after orienteering (*p* = 0.945, t = 0.301) or the 5-km run (*p* = 0.121, t = 1.918) relative to baseline.

#### 3.3.3 Bells test

Performance on the Bells test differed significantly across the three conditions (Figure 2F), as indicated by a one-way ANOVA (*F* = 34.11, *p <* 0.001, R^2^ = 0.630). *Post-hoc* comparisons showed that scores improved significantly following both orienteering (*p <* 0.001, t = 5.440) and the 5-km run (*p <* 0.001, t = 8.102) compared to baseline.

### 3.4 Correlation between cortisol and cognitive function

**Figure 3:**
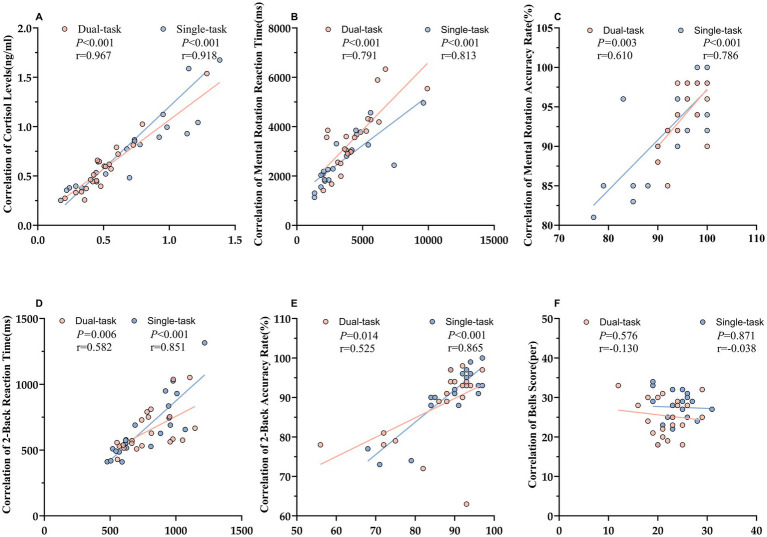
Correlations between exercise-induced cortisol changes (POST − PRE) and cognitive performance improvements. **(A)** Cortisol levels (ng·mL^−1^) at PRE and POST. **(B,C)** Mental rotation RT/ACC changes vs. cortisol changes. **(D,E)** 2-Back RT/ACC changes vs. cortisol changes. **(F)** Bells score changes vs. cortisol changes.

Spearman correlation analyses were performed to examine the associations between exercise-induced changes in cortisol levels and cognitive performance (Figure 3). Cortisol levels before and after both orienteering (r = 0.967, *p <* 0.001) and the 5-km run (r = 0.918, *p <* 0.001) were strongly correlated, confirming consistent elevations following in response to both exercise modalities.

In the mental rotation task, post-exercise cortisol changes were positively associated with RT (orienteering: r = 0.791, *p <* 0.001; 5-km run: r = 0.813, *p <* 0.001) and ACC (orienteering: r = 0.610, *p* = 0.003; 5-km run: r = 0.786, *p <* 0.001).

In the 2-Back working memory task, cortisol responses were also positively associated with RT (orienteering: r = 0.582, *p* = 0.006; 5-km run: r = 0.851, *p <* 0.001) and ACC (orienteering: r = 0.525, *p* = 0.014; 5-km run: r = 0.865, *p <* 0.001).

In contrast, no significant correlations were found between cortisol changes and performance on the Bells visual search task (orienteering: r = −0.130, *p* = 0.576; 5-km run: r = − 0.038, *p* = 0.871).

Together, these findings suggest that exercise-induced cortisol elevations following acute exercise are positively associated with cognitive improvements in tasks involving spatial processing and working memory. But not in visual search performance- highlighting a task-dependent relationship.

## 4 Discussion

This study demonstrated that cognitively enriched exercise, even when matched for physiological intensity, elicits distinct neuroendocrine and cognitive outcomes compared to traditional aerobic activity. Orienteering, but not steady-state running, led to significant elevations in salivary cortisol, suggesting that dual-task demands involving navigation, decision-making, and spatial processing effectively activate the HPA-axis. This finding aligns with recent studies showing that cognitively enriched exercise—particularly those involving coordination, spatial navigation, and decision-making—elicits stronger HPA-axis activation than endurance-only exercise, even when matched for physiological intensity (Budde et al., 2025). Functional imaging and neurobehavioral studies further suggest that dual-task training activates prefrontal and parietal regions involved in executive control and attention allocation, thereby enhancing cortisol responsiveness via top-down modulation (Surkar et al., 2025). In ecological paradigms such as virtual orienteering, these complex tasks have been shown to trigger HPA activation beyond that of physical exertion alone (Richardson and VanderKaay Tomasulo, 2022). These findings suggest that the cognitive complexity embedded in the exercise modality, rather than physical exertion alone, may be a key determinant of neuroendocrine activation.

Both exercise modalities improved RTs on tasks involving working memory (2-Back) and spatial cognition (mental rotation) relative to baseline, consistent with prior findings on acute exercise-induced cognitive enhancement (Robazza et al., 2018). However, these cognitive benefits were not reflected in the Bells visual search task, likely due to its relatively low cognitive demands and performance approaching ceiling levels, particularly in young, cognitively intact individuals (Ligeza et al., 2023). The task-dependent nature of ACC improvements highlights the selective nature of cognitive facilitation.

In contrast to complex tasks that require executive control, working memory updating, and spatial transformation—processes heavily reliant on dorsolateral prefrontal cortex (DLPFC) and hippocampal–prefrontal circuits—the Bells task involves more automatic and low-level attentional processes. These functions are less sensitive to acute physiological arousal and HPA-axis modulation, which may explain the lack of significant exercise-induced improvements in this domain (Gazzaley and Nobre, 2012; Qin et al., 2009).

Cortisol’s effects on cognitive performance appear to exhibit marked domain-specificity. Acute stress and moderate cortisol elevations preferentially modulate prefrontal cortex-dependent functions, typically enhancing cognitive performance under conditions of moderate arousal, while exerting limited influence on basic attentional processes such as those tested in the Bells task (Arnsten, 2009; Shields et al., 2016). This specificity likely explains why dual-task (orienteering) exercise led to more pronounced behavioral improvements in complex cognitive domains compared to simple visual search performance. Crucially, orienteering elicited superior cognitive enhancements relative to running, particularly in tasks demanding higher executive engagement. These findings support the hypothesis that cognitively enriched exercise modalities amplify cortisol-induced cognitive facilitation in domains supported by the prefrontal cortex and hippocampus. Our findings revealed strong positive associations between acute cortisol elevations and post-exercise improvements in cognitive performance, particularly in working memory and spatial cognition. These results align with the theory that moderate physiological stress can transiently enhance cognitive efficiency, especially in prefrontal cortex–dependent tasks (Bogdanov et al., 2021).

Recent neuroscience evidence suggests a nonlinear, dose-dependent relationship between glucocorticoid levels and prefrontal cortical function, often conceptualized as an inverted-U curve (McEwen and Morrison, 2013). At moderate levels, cortisol facilitates neuronal signaling, synaptic plasticity, and attentional tuning in the PFC and hippocampus, thereby optimizing performance on executive tasks. In contrast, excessive cortisol may suppress these circuits, impairing goal-directed cognition (Chang et al., 2015).

Moreover, moderate elevations in cortisol may enhance task performance by increasing central arousal, which facilitates attentional engagement and executive control (Li et al., 2025). This arousal-mediated enhancement is especially beneficial for cognitively demanding tasks that require sustained mental effort and top-down regulation (Sudo et al., 2022). In contrast, simpler tasks that rely more on automatic or low-level attentional processes show limited sensitivity to fluctuations in arousal (Kinomura et al., 1996; Lee et al., 2012; Sutherland and Mather, 2012). This task-dependent effect supports the view that moderate HPA axis activation promotes selective cognitive facilitation by modulating alertness rather than exerting uniform effects across all domains (Shields et al., 2019; Herman et al., 2016).

In conclusion, our findings highlight the task-dependent nature of exercise-induced cognitive enhancement and underscore the central role of cortisol as a potential mediator. Cognitive engagement during physical activity appears to augment both neuroendocrine and behavioral responses, offering a promising pathway for optimizing brain function through ecologically valid, integrated exercise interventions.

Furthermore, these findings invite future studies to adopt broader, more integrative designs that test how individual differences, task complexity, and multimodal physiological pathways jointly shape exercise-induced cognitive facilitation.

Several limitations warrant consideration. First, the present sample consisted solely of young male college students (aged 19–26 years) with prior orienteering experience, which limits the external validity of the findings. In practice, exercise participants vary widely in gender, age, and sports experience—factors that may influence the exercise–cortisol–cognition relationship. For example, hormonal responses to exercise often differ between men and women due to interactions among sex hormones, cortisol, and catecholamine systems. Future studies should therefore expand recruitment to include (i) female participants to test potential gender-specific effects, (ii) adults across broader age ranges (e.g., 26–35 years) to assess age-related variations, and (iii) individuals without prior orienteering experience to evaluate the role of task familiarity. Subgroup analyses by gender, age, and experience will clarify whether the observed dual-task exercise advantages are universal or population-specific, thereby enhancing the ecological validity of the conclusions.

Second, this study examined only immediate post-exercise cognitive performance, leaving the temporal persistence of cognitive benefits undetermined. In applied contexts—such as academic learning or workplace performance—understanding how long post-exercise facilitation lasts is essential for practical implementation. Future studies should include delayed cognitive assessments (e.g., at 1 h, 6 h, and 24 h post-exercise) synchronized with repeated salivary cortisol sampling to identify the time window of maximal cognitive benefit and its relationship with cortisol recovery kinetics. Such evidence would provide a physiological basis for recommending optimal timing of cognitively enriched exercise before demanding mental tasks.

Third, while cortisol was the primary neuroendocrine marker in this study, other physiological pathways likely contribute to the observed cognitive facilitation. Norepinephrine, which modulates arousal and attention, and brain-derived neurotrophic factor (BDNF), which supports synaptic plasticity, may interact with cortisol to influence executive function. In addition, neurophysiological tools such as functional near-infrared spectroscopy (fNIRS) or electroencephalography (EEG) could be applied to characterize prefrontal activation patterns during and after dual-task exercise. Incorporating these multimodal measures would enable construction of an integrative “exercise type → multi-physiological pathway → cognitive performance” model, thereby clarifying whether dual-task advantages arise from cortisol alone or from coordinated effects across neuroendocrine and neural systems.

Fourth, the present dual-task condition used a single orienteering complexity (27 checkpoints), precluding analysis of dose–response relationships between cognitive load, cortisol response, and cognitive performance. Individuals differ in cognitive capacity and tolerance to task complexity; hence, future studies should include dual-task conditions with graded cognitive loads (e.g., 15, 27, and 40 checkpoints) while maintaining matched physical intensity. This would allow mapping of the optimal cognitive load for maximizing benefits and help tailor exercise prescriptions for diverse populations, such as low-load dual-task exercise for beginners or older adults and high-load tasks for young, cognitively intact individuals.

Together, these refinements will broaden the generalizability, mechanistic insight, and practical applicability of future research on cognitively enriched exercise and its neuroendocrine–cognitive interactions.

## 5 Conclusion

Orienteering, but not steady-state running, elicited significant cortisol elevations and was linked to greater improvements in executive-related tasks. Both exercise modes enhanced reaction times, yet accuracy remained unchanged, indicating a speed-focused facilitation. The consistent cortisol–cognition coupling observed only in dual-task exercise underscores the task-dependent nature of acute exercise-induced cognitive benefits. These findings suggest that integrating cognitive demands into exercise may be an effective strategy to optimize neuroendocrine activation and executive function.

## Data Availability

The raw data supporting the conclusions of this article will be made available by the authors, without undue reservation.

## References

[ref1] Arnsten A. F. T. (2009). Stress Signalling pathways that impair prefrontal cortex structure and function. Nat. Rev. Neurosci. 10, 410–422. doi: 10.1038/nrn2648, 19455173 PMC2907136

[ref2] Basso J. C. Suzuki W. A. (2017). The effects of acute exercise on mood, cognition, neurophysiology, and&nbsp;neurochemical pathways: a review. Brain Plasticity 2, 127–152. doi: 10.3233/BPL-160040, 29765853 PMC5928534

[ref3] Becker L. Kaltenegger H. C. Nowak D. Rohleder N. Weigl M. (2023). Differences in stress system (re-)activity between single and dual- or multitasking in healthy adults: a systematic review and Meta-analysis. Health Psychol. Rev. 17, 78–103. doi: 10.1080/17437199.2022.2071323, 35477383

[ref4] Bermejo J.-L. Valldecabres R. Villarrasa-Sapiña I. Monfort-Torres G. Marco-Ahulló A. Couto B. R. D. (2022). Increased cortisol levels caused by acute resistance physical exercise impair memory and learning ability. PeerJ 10:e13000. doi: 10.7717/peerj.13000, 35345590 PMC8957269

[ref5] Bogdanov M. Nitschke J. P. LoParco S. Bartz J. A. Otto A. R. (2021). Acute psychosocial stress increases cognitive-effort avoidance. Psychol. Sci. 32, 1463–1475. doi: 10.1177/09567976211005465, 34464216

[ref6] Budde H. Machado S. Ribeiro P. Wegner M. (2015). The cortisol response to exercise in young adults. Front. Behav. Neurosci. 9:13. doi: 10.3389/fnbeh.2015.00013, 25691863 PMC4315045

[ref7] Budde H. Voelcker-Rehage C. Pietraßyk-Kendziorra S. Ribeiro P. Tidow G. (2008). Acute coordinative exercise improves attentional performance in adolescents. Neurosci. Lett. 441, 219–223. doi: 10.1016/j.neulet.2008.06.024 18602754

[ref8] Budde H. Wegner M. Ahrens C. Velasques B. Ribeiro P. Machado S. . (2025). Effects of acute coordinative vs. endurance exercise on cortisol concentration in healthy women and men. Sports Med. Open 11:72. doi: 10.1186/s40798-025-00884-z, 40494957 PMC12151962

[ref9] Caplin A. Chen F. S. Beauchamp M. R. Puterman E. (2021). The effects of exercise intensity on the cortisol response to a subsequent acute psychosocial stressor. Psychoneuroendocrinology 131:105336. doi: 10.1016/j.psyneuen.2021.105336, 34175558

[ref10] Chang Y.-K. Chu C.-H. Wang C.-C. Wang Y.-C. Song T.-F. Tsai C.-L. . (2015). Dose–response relation between exercise duration and cognition. Med. Sci. Sports Exerc. 47, 159–165. doi: 10.1249/MSS.0000000000000383, 24870572

[ref11] Davis P. A. Sörman D. Carlberg A. Rognsvåg E. Stenling A. (2022). The psychophysiological influence of exertion and affect on sport-specific cognitive and physical performance. J. Sci. Med. Sport 25, 764–769. doi: 10.1016/j.jsams.2022.05.008, 35697601

[ref12] Gallou-Guyot M. Mandigout S. Bherer L. Perrochon A. (2020). Effects of Exergames and cognitive-motor dual-task training on cognitive, physical and dual-task functions in cognitively healthy older adults: an overview. Ageing Res. Rev. 63:101135. doi: 10.1016/j.arr.2020.101135, 32768594

[ref13] Garber C. E. Blissmer B. Deschenes M. R. Franklin B. A. Lamonte M. J. Lee I.-M. . (2011). Quantity and quality of exercise for developing and maintaining cardiorespiratory, musculoskeletal, and Neuromotor fitness in apparently healthy adults: guidance for prescribing exercise. Med. Sci. Sports Exerc. 43, 1334–1359. doi: 10.1249/MSS.0b013e318213fefb, 21694556

[ref14] Gazzaley A. Nobre A. C. (2012). Top-down modulation: bridging selective attention and working memory. Trends Cogn. Sci. 16, 129–135. doi: 10.1016/j.tics.2011.11.014, 22209601 PMC3510782

[ref15] Gronwald T. De Bem Alves A. C. Murillo-Rodríguez E. Latini A. Schuette J. Budde H. (2019). Standardization of exercise intensity and consideration of a dose–response is essential. Commentary on “exercise-linked FNDC5/Irisin rescues synaptic plasticity and memory defects in Alzheimer’s models”, by Lourenco et al., published 2019 in nature medicine. J. Sport Health Sci. 8, 353–354. doi: 10.1016/j.jshs.2019.03.006, 31333889 PMC6620472

[ref16] Gronwald T. Velasques B. Ribeiro P. Machado S. Murillo-Rodríguez E. Ludyga S. . (2018). Increasing exercise’s effect on mental health: exercise intensity does matter. Proc. Natl. Acad. Sci. USA 115, E11890–E11891. doi: 10.1073/pnas.1818161115, 30568027 PMC6304946

[ref17] Hawley J. A. Noakes T. D. (1992). Peak power output predicts maximal oxygen uptake and performance time in trained cyclists. Europ. J. Appl. Physiol. 65, 79–83. doi: 10.1007/BF01466278, 1505544

[ref18] Herman J. P. McKlveen J. M. Ghosal S. Kopp B. Wulsin A. Makinson R. . (2016). Regulation of the hypothalamic-pituitary-adrenocortical stress response. Compr. Physiol. 6, 603–621. doi: 10.1002/cphy.c150015, 27065163 PMC4867107

[ref19] Kinomura S. Larsson J. Gulyas B. Roland P. E. (1996). Activation by attention of the human reticular formation and thalamic Intralaminar nuclei. Science 271, 512–515. doi: 10.1126/science.271.5248.512, 8560267

[ref20] Koutsandréou F. Wegner M. Niemann C. Budde H. (2016). Effects of motor versus cardiovascular exercise training on children’s working memory. Med. Sci. Sports Exerc. 48, 1144–1152. doi: 10.1249/MSS.0000000000000869, 26765631

[ref21] Kraemer R. R. Kraemer B. R. (2023). The effects of peripheral hormone responses to exercise on adult hippocampal neurogenesis. Front. Endocrinol. 14:1202349. doi: 10.3389/fendo.2023.1202349, 38084331 PMC10710532

[ref22] Lee T.-H. Itti L. Mather M. (2012). Evidence for arousal-biased competition in perceptual learning. Front. Psychol. 3:241. doi: 10.3389/fpsyg.2012.00241, 22833729 PMC3400437

[ref23] Li R.-H. Chen T.-R. Gilson N. D. Brazaitis M. Cheng Y.-T. Wu H.-F. . (2025). Acute concurrent exercise improves inhibitory control without mediating the role of lactate: an event-related potential study. Sports Med - Open 11:12. doi: 10.1186/s40798-024-00809-2, 39871026 PMC11772916

[ref24] Ligeza T. S. Vens M. J. Bluemer T. Junghofer M. (2023). Acute aerobic exercise benefits allocation of neural resources related to selective attention. Sci. Rep. 13:8624. doi: 10.1038/s41598-023-35534-5, 37244926 PMC10220342

[ref25] Lunardini F. Luperto M. Romeo M. Basilico N. Daniele K. Azzolino D. . (2020). Supervised digital neuropsychological tests for cognitive decline in older adults: usability and clinical validity study. JMIR Mhealth Uhealth 8:e17963. doi: 10.2196/17963, 32955442 PMC7536607

[ref26] Lupien S. J. McEwen B. S. Gunnar M. R. Heim C. (2009). Effects of stress throughout the lifespan on the brain, behaviour and cognition. Nat. Rev. Neurosci. 10, 434–445. doi: 10.1038/nrn2639, 19401723

[ref27] McEwen B. S. Morrison J. H. (2013). The brain on stress: vulnerability and plasticity of the prefrontal cortex over the life course. Neuron 79, 16–29. doi: 10.1016/j.neuron.2013.06.028, 23849196 PMC3753223

[ref28] Pellegrini-Laplagne M. Dupuy O. Sosner P. Bosquet L. (2022). Acute effect of a simultaneous exercise and cognitive task on executive functions and prefrontal cortex oxygenation in healthy older adults. Brain Sci. 12:455. doi: 10.3390/brainsci12040455, 35447987 PMC9027217

[ref29] Qin S. Hermans E. J. Luo J. Fernández G. (2009). Acute psychological stress reduces working memory-related activity in the dorsolateral prefrontal cortex. Biol. Psychiatry 66, 25–32. doi: 10.1016/j.biopsych.2009.03.006, 19403118

[ref30] Richardson A. E. VanderKaay Tomasulo M. M. (2022). Stress-induced HPA activation in virtual navigation and spatial attention performance. BMC Neurosci. 23:40. doi: 10.1186/s12868-022-00722-y, 35764937 PMC9241311

[ref31] Robazza C. Izzicupo P. D’Amico M. A. Ghinassi B. Crippa M. C. Di Cecco V. . (2018). Psychophysiological responses of junior orienteers under competitive pressure. PLoS One 13:e0196273. doi: 10.1371/journal.pone.0196273, 29698498 PMC5919653

[ref32] Russell G. Lightman S. (2019). The human stress response. Nat. Rev. Endocrinol. 15, 525–534. doi: 10.1038/s41574-019-0228-0, 31249398

[ref33] Shields G. S. Rivers A. M. Ramey M. M. Trainor B. C. Yonelinas A. P. (2019). Mild acute stress improves response speed without impairing accuracy or interference control in two selective attention tasks: implications for theories of stress and cognition. Psychoneuroendocrinology 108, 78–86. doi: 10.1016/j.psyneuen.2019.06.001, 31229636 PMC6707871

[ref34] Shields G. S. Sazma M. A. Yonelinas A. P. (2016). The effects of acute stress on Core executive functions: a Meta-analysis and comparison with cortisol. Neurosci. Biobehav. Rev. 68, 651–668. doi: 10.1016/j.neubiorev.2016.06.038, 27371161 PMC5003767

[ref35] Sudo M. Costello J. T. McMorris T. Ando S. (2022). The effects of acute high-intensity aerobic exercise on cognitive performance: a structured narrative review. Front. Behav. Neurosci. 16:957677. doi: 10.3389/fnbeh.2022.957677, 36212191 PMC9538359

[ref36] Surkar S. M. Lin C.-C. Trotter B. Phinizy T. Sylcott B. (2025). Effects of dual-task training on cognitive-motor learning and cortical activation: a non-randomized clinical trial in healthy young adults. PLoS One 20:e0322036. doi: 10.1371/journal.pone.0322036, 40338969 PMC12061167

[ref37] Sutherland M. R. Mather M. (2012). Negative arousal amplifies the effects of saliency in short-term memory. Emotion 12, 1367–1372. doi: 10.1037/a0027860, 22642352 PMC3586810

[ref38] Suwabe K. Byun K. Hyodo K. Reagh Z. M. Roberts J. M. Matsushita A. . (2018). Rapid stimulation of human dentate gyrus function with acute mild exercise. Proc. Natl. Acad. Sci. USA 115, 10487–10492. doi: 10.1073/pnas.1805668115, 30249651 PMC6187140

[ref39] Wilke J. Vogel O. Ungricht S. (2020). Can we measure perceptual-cognitive function during athletic movement? A framework for and reliability of a sports-related testing battery. Phys. Ther. Sport Off. J. Asso. Chartered Physiotherapists Sports Med. 43, 120–126. doi: 10.1016/j.ptsp.2020.02.016, 32145687

[ref40] Wohlwend M. Olsen A. Håberg A. K. Palmer H. S. (2017). Exercise intensity-dependent effects on cognitive control function during and after acute treadmill running in young healthy adults. Front. Psychol. 8:406. doi: 10.3389/fpsyg.2017.00406, 28377735 PMC5359239

[ref41] Wunsch K. Wurst R. von Dawans B. Strahler J. Kasten N. Fuchs R. (2019). Habitual and acute exercise effects on salivary biomarkers in response to psychosocial stress. Psychoneuroendocrinology 106, 216–225. doi: 10.1016/j.psyneuen.2019.03.015, 31003138

